# Fortification of cocoa semi-skimmed milk formulations with native lactic acid bacteria: Cell viability, physicochemical and functional properties for developing novel foods

**DOI:** 10.3389/fnut.2022.1008871

**Published:** 2022-10-13

**Authors:** Gabriela N. Tenea, Pamela Ascanta

**Affiliations:** Biofood and Nutraceutics Research and Development Group, Faculty of Engineering in Agricultural and Environmental Sciences, Universidad Técnica del Norte, Ibarra, Ecuador

**Keywords:** cocoa, lactic acid bacteria, antioxidant activity, polyphenols, probiotics

## Abstract

This study aimed to evaluate several cocoa semi-skimmed milk formulations (CSMFs) as potential carriers of native lactic acid bacteria (LAB) strains to obtain novel probiotic beverages (PBs) with improved technological and functional characteristics, and satisfactorily organoleptic acceptance. The viability of two native LAB (*Lactiplantibacillus plantarum* UTNGt2 and *Lactiplantibacillus pentosus* UTNGt5) was assessed in comparison with two references (*Lactococcus lactis* subsp. *lactis* ATCC11474 and *Limosilactobacillus reuteri* DSM17938) strains in supplemented CSMFs throughout storage with refrigeration. The optimum conditions to produce novel beverages supplemented with native LAB were pH 6.6, 42°C, and 1 h of fermentation. Moreover, the effect of LAB strains fortification on pH, titratable acidity, total solids (°Brix), total polyphenolic compounds (TPC), antioxidant capacity (AOX), and ascorbic acid content (AAC), total proteins and fat, at initial and final storage was evaluated. The addition of two native LAB strains did alter the physicochemical quality of CSMFs to a lesser extent, where the bioactive molecules improved significantly (*p* < 0.05) with the increase of cocoa concentration and depending on the supplied strain. Although a statistically significant (*p* < 0.05) decrease in cell counts was recorded during storage, the LAB cells were found to be viable up to 21 days of storage at 4°C (>6 logCFU/ml), which is sufficient in number to prove their stability *in vitro*. Overall organoleptic results suggested that LAB supplementation had a significant impact on sensory attributes with satisfactory acceptability (>78%) of PBs containing the native strains and 1–2% cocoa, while CSMFs counterparts were less appreciated (40%) as perceived off-flavor. It appears that supplying bacteria to CSMF preserves flavor in the final product. Furthermore, the final beverages were free of harmful bacteria; thus, they comply with consumer safety regulations. This study concludes that CSMF can be used as a carrier of native LAB strains, maintaining cell viability, unaltered physicochemical properties, and improved functional and sensory characteristics, for which final beverages can be regarded as functional food. From the application standpoint, these formulations are an alternative to delivering native LAB strains and could help the cocoa and dairy industry to develop more attractive products for the growing regional market.

## Introduction

In the food industry, trends in the consumption of healthy foods increased, therefore it is of interest to develop novel products with enhanced nutritional and pharmacological characteristics. LAB are used in food production because they provide taste, odor, and texture characteristics that are pleasing to the consumer (1). Currently, bacteria of genus *Lactobacillus* are used for the preparation of novel functional foods, their presence in the gastrointestinal tract being considered beneficial (2). Several studies indicated that these bacteria may contribute to the balance of the intestinal microbiota of the host and increase immunity, have antimicrobial effect against pathogenic bacteria, antiviral effects, are resistant to a wide range of pH and temperature, and are also thought to optimize digestion and milk tolerance (3). In addition, these microorganisms, known as probiotics, are active ingredients to improve the health of diabetic and non-diabetic consumers (4).

Various dairy products are considered ideal carriers for probiotic administration in the human digestive tract (5), while there is also a growing demand for non-dairy food matrices for probiotic transfer. Thus, researchers seek for developing probiotic products from different substrates, such as fruits-based raw materials (6). Fruits substrates have the virtue of containing useful nutrients such as minerals, vitamins, fiber, and antioxidants. However, successful habituation of probiotic microorganisms in these materials is strain-, and food matrix-dependent (7). A recent investigation showed that cocoa powder or chocolate could be a suitable vehicle for the delivery of probiotics (8, 9). Due to the high content of carbohydrates, cocoa powder is considered the cheapest material for an effective encapsulating agent to deliver probiotics (9). More recently, a probiotic chocolate milk containing *Lacticaseibacillus rhamnosus* CRL1505 showed high efficacy in the prevention of gastrointestinal and respiratory infections, two very prevalent problems in childhood (10). MojoMoo is the first product on the market and the first chocolate drink mix to incorporate probiotic *Bacillus coagulans* (11). It is classified as a functional food containing less sugar than regular chocolate milk mixes and contains 10 times the active probiotic cultures than a probiotic yogurt. In addition, Peruvian cocoa beans and kefir shake contained live “*ryazhanka*” cultures (fermented milk), tapioca starch, and concentrated lemon; further including *Bifidobacterium, Lactobacillus acidophilus, Lactiplantibacillus plantarum, Lacticasei. casei, L. rhamnosus* (12). Thus, the manufacturing of novel beverages containing cocoa and fortified with probiotics can be healthy and attractive functional foods for health-conscious consumers.

Ecuador is one of the most known providers of cocoa (*Theobroma cacao* L.) powder worldwide as the raw material for chocolate (13). About 70% of the production is considered “fine” and “aroma” cocoa and was denominated “superior flavor” cocoa. These cocoa is produced by Criollo and Trinitario (hybrids) varieties and represent approximately 8% of the total world harvest (14). The pulp is a sweet white mucilaginous layer that sits next to the seeds of the fruit. In the cocoa pulp structure, 82–87% moisture, 10–15% sucrose, 1–3% citric acid, and 1–1.5% pectin. In addition, proteins, amino acids, vitamins (mainly vitamin C), and minerals remain present, making this pulp a rich medium for microbial growth (15, 16). Cocoa beans are used to produce cocoa powder carrying a strong load of antioxidants, known for their antiatherogenic, antiradical, and anticancer properties (17, 18). Early research indicated that the variety “aroma fina” originated from the Ecuadorian coast has high content epicatechin (19), a primary flavonoid to exhibit health-promoting biological activity (20).

Considering the extensive changes brought about by Ecuadorian government policies, various underdeveloped natural areas such as subtropical forests, reservoir of medicinal plants and fruits, have been considered as important genetic resources that can be used for biotechnological research. Nonetheless, the Ecuadorian market is limited to processed products containing cocoa or probiotics. Cocoa is mostly exported or sold as powder at the national level, while the probiotic products are restricted to yogurts containing the commercial *Lactobacillus* LGG strain. Aiming at developing new probiotic products containing native LAB strains, we proposed to take advantage of the nutritional organic cocoa “aroma fina” as a delivery raw material for a beneficial selected native lactic acid bacteria originated from native niches and showing *in vitro* probiotic-like capacity to obtain a novel flavored beverage supplemented with native strains. This approach will help in the future expand the processed products in the local market and contribute to consumer demand for nutritious foods with health benefits.

In previous research, the whole genome of *L. plantarum* strain UTNGt2 originated from wild white cocoa fruits, and their probiotic and antimicrobial potential *in vitro* was described (21). These data were complemented by genomics and stability assessment, as suggested by previous method guidelines (22). Thus, the genome analysis showed no plasmid, no virulence genes, antibiotic resistance, and biogenic amine-related genes (i.e., lysine decarboxylase, ornithine/lysine-, and arginine decarboxylase, spermidine synthase, arginase, histidine, tyrosine decarboxylase, and tryptophan decarboxylase, were detected through a complete genome sequencing study. Furthermore, *Lactiplantibacillus pentosus* strain UTNGt5 (same origin as UTNGt2) showed high resistance to synthetic gastric acid (pH 2.5), bile salts (0.3%), tolerance to different sodium chloride concentrations (1, 3, and 5%), and growth conditions (15 and 45°C) and was also tested hereafter for its technological and functional properties. However, this study evaluated the effect of CSMF containing different concentrations of organic cocoa powder (1, 2, and 3%) as a vehicle of two native LAB and two reference strains to produce novel cocoa semi-skimmed milk-based beverages supplemented with native LAB strains with acceptable flavor and improved functional characteristics. Two methods: (a) fortification of CSMF independently with four LAB strains and direct storage with refrigeration (no fermentation step), and (b) fortification of CSMF with the target strains followed by 1 h fermentation step) were compared for beverage production and the effect on cell viability during storage coupled with the sensorial properties (acidity, taste, aspect, sweetness, and overall impression) was evaluated to select the optimal conditions of beverage manufacturing. Moreover, the functional molecules (TPC, AOX, and AAC) and the technological properties (pH, titratable acidity, total solids, proteins, and fat) were evaluated at initial (1) and last (21) days of storage. However, developing a novel beverage cocoa-base supplemented with native LAB strains might be an advantage to increase the market of functional products having similar or improved characteristics than the imported probiotic counterparts. Besides, the addition of cocoa to the milk is said to add flavor to the product and give it a nutraceutical value.

## Materials and methods

### Bacterial strain and growth conditions

Two native strains, *L. plantarum* strain UTNGt2 (GenBank accession no. KY041688.1) and *L. pentosus* strain UTNGt5 (GenBank accession no. ON307470) were previously isolated from wild white cocoa fruits (*Theobroma grandiflorum*) (21). Two commercial strains, *Lactococcus lactis* subsp. *lactis* ATCC11454 (L.Lac) and *Limosilactobacillus reuteri* DSM17938 (L.r), were used for comparison. The strains were maintained as frozen stock cultures in MRS broth (Difco, Detroit, MI, USA) containing 20% (v/v) glycerol. The chemicals and reagents used were of analytical grade.

### Manufacturing of PBs

#### Raw materials and preparation of cocoa semi-skimmed milk formulations

Organic cocoa powder (fine-flavor or “aroma fina”) originated from Manabi Province (Ecuadorian coast) and semi-skimmed milk (UHT) were purchased from the local market. A stock cocoa solution (15%) was prepared by diluting the powder in sterile water, sterilizing for 5 min at 100°C and then filtering to remove any residual particles. The semi-skimmed milk was sterilized for 12 min at 100°C to provide lactic acid bacteria-free skim milk (23). CSMFs were prepared as follows: (a) CSMF1: semi-skimmed milk with 1% cocoa + 2% glucose; (b) CSMF2: semi-skimmed milk with 2% cocoa + 2% glucose; (c) CSMF3: semi-skimmed milk with 3% cocoa + 2% glucose; and (d) CSMF4: semi-skimmed milk + 2% glucose without added cocoa. These formulations were distributed in sterile jars prior to independently inoculation with each bacterial strain.

#### Cell inoculum and fermentation conditions

Lactic acid bacteria strains were routinely activated and sub-cultured in De Man, Rogosa, and Sharpe (MRS) broth (Difco, Detroit, MI, USA) under anaerobic conditions at 37°C. Bacterial precultures (5 ml) were transferred to 100 ml flasks of MRS broth and incubated for 24 h at 37°C to obtain inoculum biomass; cells were harvested by centrifugation (5000 x rpm for 5 min at 4°C) and washed once with sterile 0.1 M sodium phosphate buffer at pH 7.0. Each bacterial culture of 5.4 grams cells (7.5 logCFU/ml cell density), as explained above, was inoculated into 750 ml of each formulation described above.

A total of 16 combinations (PB1–PB16) were obtained by 2 procedures: (a) strains supplementation without fermentation step (0 h) and direct storage at 4°C; (b) strains supplementation with 1 h of fermentation at 42°C (statically in a water bath). Each of the bottles was cooled to 5°C for 30 min and the pH was completed (Supplementary Table 1), then adjusted to 6.60 with sterile bicarbonate solution (10%); the result of this method was considered day 0. The bottles were stored in refrigeration for 21 days and the viability, titratable acidity, pH, total solids, and viability of the inoculated probiotic bacteria were determined at initial and final storage. All experiments were conducted in triplicate, starting with a new batch of CSMF. As controls, the following combinations were used: SM1: semi-skimmed milk + 1% sterile distilled water + 2% glucose; SM2: semi-skimmed milk + 2% sterile distilled water + 2% glucose; SM3: semi-skimmed milk + 3% sterile distilled water + 2% glucose.

### Evaluation of the physicochemical properties of raw materials, cocoa semi-skimmed milk formulations, and PBs

pH, titratable acidity, and total soluble solids (°Brix) were determined in the raw materials (sterile cocoa solutions, UHT, and autoclaved semi-skimmed milk), cocoa-free semi-skimmed milk (SMs), CSMFs, and PBs. The pH was measured by electrode immersion with a pH meter (S210, Mettler Toledo, Columbus, OH, USA). The titratable acidity was determined by titrating 10 ml of each PB with 0.1 N NaOH using phenolphthalein as an indicator [(24): NTE INEN 0013/1984]. Results were expressed as a percentage of lactic acid (milk and mix cocoa semi-skimmed milk) or citric acid (cocoa solution) per 100 ml of product [(25): FAO CXS: 243-2003]. Following the same procedure, PBs were evaluated during storage on days 1, 7, 14, and 21. Similarly, the total solids in the solution was determined using a digital refractometer (26). Each of the measurements was carried out in triplicate using different batches of raw material.

### Determination of cell viability throughout storage

The cell viability was determined in all beverages obtained by both methods at the initial (1) and final (21) days of storage with refrigeration, to select for the optimum method that comply with the main criteria of maintaining the viability above limit (27), and liquid status throughout the storage. Alike, the cell viability was determined in beverages obtained with the optimized method at intervals of 1 week (1, 7, 14) for 21 days. One milliliter of each beverage obtained was diluted in 9 ml of sterile peptone (Merck, Darmstadt, Germany), water (0.1%), and the appropriate dilutions were plated on MRS agar (Difco, Detroit, MI, USA) for enumeration of total LAB in each beverage combination (28). Colony-forming units (CFU) per ml of beverage were recorded for plates containing 50–350 colonies. The CFU/ml was determined automatically by scanning the plates with a microplate reader (SCAN500, Interscience, France). Each of the measurements was carried out in triplicate using different batches of raw material.

### Determination of total protein and fat of PBs at initial and final storage

The total protein content was determined by the Kjeldahl method according to the Ecuadorian regulation [(29): NTE INEN 2609/2012]. In brief, the method determines the total nitrogen content in the sample, and the nitrogen percentage is converted to crude protein by multiplying by 6.25 (26). The Gerber method was used as an indicator for the fat content in semi-skimmed milk (no cocoa added), and total solids were determined by oven drying [(30): NTE INEN 0012/1978]. For the raw cocoa (10% solution) and PBs the Soxhlet method was used (31, 32). Each of the measurements was carried out in triplicate using different batches of raw materials. Besides, the total protein and fat (%) were estimated in the CSMFs matrices prior to bacteria inoculation.

### Functional analysis of raw materials, cocoa semi-skimmed milk formulations, and PBs

Total polyphenolic compounds (TPC), antioxidant capacity (AOX), and ascorbic acid content (AAC) were determined in the raw material (sterile cocoa solutions, UHT and autoclaved semi-skimmed milk), SMs, CSMFs, and PBs, at the initial (day 1) and final steps of storage (day 21). Each of the measurements was carried out in triplicate using different batches of raw material.

#### Total polyphenolic compounds analysis

Determination of TPC was performed using the Folin–Ciocalteu method with gallic acid (Sigma-Aldrich Co. LLC, Saint Louis, MO, USA) as standard, as previously described (33). The supernatant was collected after the treatment of each sample extract (5 ml) with methanol 98% (10 ml) and centrifuged at 8000 x *g* for 20 min. The supernatants were filtered through a 0.45 μM hydrophilic filter (ANPEL Scientific Instrument Co., Shanghai, China) and used as extract (500 μl) for determining the phenolic content at days 1 and 21 of storage. Absorbance at 715 nm was measured using a spectrophotometer (Jenway 6705 UV/Vis, Bibby Scientific Limited, ST15 OSA, UK) and the graphical dependence of solution absorbance on the amount of gallic acid was plotted. The results were reported as equivalent milligrams of gallic acid (GAE)/L of product. The analyses were carried out in triplicate starting with a new batch of samples (three extracts). The result was calculated from nine repetitions.

#### Antioxidant capacity activity

Antioxidant activity was determined using the DPPH (1, 1-diphenyl-2-picryl-hydrazyl, Sigma-Aldrich Co. LLC, Saint Louis, MO, USA) radical scavenging activity method as previously described (34). The supernatant was collected after treatment of each sample extract (5 ml) with ethanol 98% (10 ml) and centrifuged at 8000 x *g* for 20 min. 100 μl of PB extract from each beverage was mixed with 2.9 ml of methanolic solution of DPPH (0.045 mg/ml). Absorbance was measured at 517 nm using an ultraviolet spectrophotometer (Jenway 6705 UV/Vis, Bibby Scientific Limited, ST15 OSA, UK) after the mixture was kept in the dark for 30 min. The relative antioxidant in a mixture sample to scavenge DPPH was compared with a Trolox standard (Sigma-Aldrich Co., LLC, Saint Louis, MO, USA). The results were expressed in equivalent μmol Trolox/L. The analyses were carried out in triplicate starting with a new batch of samples (three extracts). The result was calculated from nine repetitions.

#### Ascorbic acid content determination

Vitamin C (reduced ascorbic acid) content was determined on days 1 and 21 of storage following a standard 2,6 dichloroindophenol titrimetric method (26). As a standard, L-ascorbic acid (1 mg/ml) was used, and the concentration was calculated by comparison with the standard and expressed as equivalent mg acid ascorbic/L. The analyses were carried out in triplicate starting with a new batch of samples (three extracts). The result was calculated from nine repetitions.

### Microbial safety of the final products

Before sensorial analysis, the microbiological safety (total coliforms, presence of *Salmonella/Shigella*, *Escherichia coli*, yeasts, and molds) was performed using the plate count agar method to ensure that no harmful microorganisms were present in the obtained PB products (35). Detection of total coliforms and *E. coli* was performed in chromocult agar (Difco, Detroit, MI, USA) at 24 h of incubation at 37°C. Fungi and molds were detected in potato dextrose peptone agar after 5 days of incubation at 25°C. *Salmonella* spp. counts were determined in SS (Shigella-Salmonella) agar medium.

### Sensorial analysis

The sensory properties were evaluated based on a descriptive characteristic of beverages obtained by the two methods (with and without fermentation step) by trained panelists (*n* = 15) recruited from the laboratory staff and students at the Technical University of the North. SMs and CSMFs maintained under the same storage conditions (21 days) were used as controls. Subjects received 10 ml of each product in 100 ml beakers at room temperature. They were asked to compare the drinks and indicate if they differed from a sensory point of view: acidic taste (with 5 points hedonic scale, where 5 represents the highest acidity and 1, no acidity), sweetness (with 5 points hedonic scale, where 5 represents the highest sweetness and 1, no sweet), cocoa smell (with 4 points hedonic scale, where 4 represents the highest cocoa smell and 1, no smell), cocoa aroma (acceptable), or no aroma (no-acceptable). The texture was also appreciated as liquid and viscous (yogurt texture). Those scored parameters were considered as the “overall impression” of the beverage recorded as (–) not acceptable/(–) off-flavor, (+) acceptable/(+) pleasant aroma.

### Statistical analysis

Results were reported as mean ± standard error. The normal data distribution was employed with Shapiro–Wilk test [RStudio Version 1.2.1335 (36) Inc., Boston, MA, USA, 2020]. The effect of procedure (fermentation vs. no-fermentation) on the cell viability, the ANOVA (SPSS 13.0, Inc., Chicago, IL, USA) with a split-split-plot experimental design was performed. Then, Duncan’s multiples tests and LSD (Least Significant Difference with Bonferroni correction) were applied to determine significant differences between the means. One-way ANOVA and Tukey’s test for comparison of means were performed to determine significant differences (*p* < 0.05) in pH, acidity, and total soluble solids (°Brix). Besides PCA analysis was conducted on the total protein, fat, pH, °Brix, and acidity, in PBs and CSMFs at the end of storage. Furthermore, to evaluate the effect of bacteria on physicochemical and functional parameters a PCA analysis was conducted on 8 variables (pH, acidity, °Brix, AOX, TPC, AAC, fat, and protein) at the end of storage. Additionally, PCA was conducted on nine variables (cell counts, pH, acidity, °Brix, AOX, TPC, AAC, fat, and protein) determinations using the correlation matrix on days 1 and 21 of storage.

## Results and discussion

### Selection of raw materials for cocoa semi-skimmed milk formulation production: Physicochemical and functional characteristics

The development of a novel cocoa semi-skimmed milk-based beverage fortified with native LAB strains with probiotic potential with acceptable flavor and optimization of beneficial molecules depends on the matrix composition, the cultivar strength, and the capacity to remain in a sufficient amount in the newly matrix, as well as the procedure applied for the manufacturing of the final beverage. While there were several differences between non-sterile whole milk (purchased from a farm) and commercial UHT whole milk, the raw materials, semi-skimmed milk, and cocoa, comply with the national regulatory requirements [(37): NTE INEN 701: 2009; (38): NTE INEN 620: 2017] (Supplementary Table 2). Based on the results, the physicochemical characteristics of CSMFs vary with the matrix composition and storage time (Table 1). A statistically significant (*p* < 0.05) increase in pH and acidity was observed at the end of storage (day 21st) in both CSMFs and SMs counterpart, indicating that storage can influence the relationship between matrix elements such as lactose, protein, and sugars, in the mix (cocoa and semi-skimmed milk) and semi-skimmed milk with glucose. These results agreed with early reports indicating that storage could influence the physicochemical and functional characteristics of milk (39). A low concentration of ascorbic acid was found in UHT, autoclaved semi-skimmed milk, and SMs, while the highest concentration was detected in the sterile 3% cocoa solution (Supplementary Table 2). The addition of cocoa to the SMs results in the vitamin C increase, suggesting that cocoa may contribute to the overall ascorbic acid content in the CSMFs (Table 1). During autoclavation, some Millard reactions are likely to occur with a direct impact on vitamin C content. Early research showed that the early or late Maillard reactions were detected in bottle sterilized milk samples compared with UHT samples, and in fortified milk samples compared with cow’s milk, which resulted in changes in vitamin C content (40). Nonetheless, in this study, no impact on vitamin C degradation was detected during storage (day 21) in the raw materials, SMs and CSMFs (Supplementary Figure 1). Similarly, statistically significant (*p* < 0.05) increase in TPC and AOX was detected with the increase of the cocoa concentration, indicating the positive impact of cocoa on the functional characteristics of the formulations (Table 1). Likewise, a statistically significant (*p* < 0.05) difference in the antioxidant capacity was observed within the raw materials, with the 3% cocoa solution having the highest concentration (Supplementary Table 2). Early research indicated that the incorporation of cocoa seed extract in milk beverages containing coffee results in increased antioxidant capacity (41). Nonetheless, in our study, at the end of storage, the antioxidant activity decreases by about 15% in all CSMF (Supplementary Figure 1). Recent studies showed that the storage temperature and geographical origin of cocoa beans influenced the overall antioxidant capacity and polyphenols in cocoa-supplemented dairy drinks (18, 42, 43). Taken together, the results suggested that cocoa contributes to the overall antioxidant activity, polyphenols, and vitamin C content in the semi-skimmed milk cocoa matrix and varies with the concentration of cocoa supplied in the matrix and the storage period.

**TABLE 1:** Effect of cocoa on physicochemical and functional characteristics of the CSMFs at initial (1) and final (21) day of storage.

Samples	Storage time (days)	pH	Total solids (°Brix)	Acidity	AOX (μM Trolox/L)	TPC (mgGAE/L)	AAC (mg/L)
CSMF1	1	6.59 ± 0.01^bA^	11.27 ± 0.01^bB^	0.17 ± 0.01^aA^	3827.26 ± 0.01^aC^	23.25 ± 0.01^aC^	0.45 ± 0.01^aB^
	21	6.71 ± 0.01^aA^	11.60 ± 0.01^aA^	0.13 ± 0.01^bB^	3271.33 ± 0.01^bF^	17.59 ± 0.01^bE^	0.45 ± 0.01^aB^
CSMF2	1	6.60 ± 0.01^aA^	11.30 ± 0.01^bB^	0.17 ± 0.01^aA^	4025.04 ± 0.01^aB^	24.89 ± 0.01^aB^	0.53 ± 0.01^aAB^
	21	6.69 ± 0.01^aA^	11.50 ± 0.01^aA^	0.13 ± 0.01^bB^	3472.81 ± 0.01^bE^	17.78 ± 0.01^bE^	0.53 ± 0.01^aAB^
CSMF3	1	6.59 ± 0.01^bA^	11.40 ± 0.01^aAB^	0.17 ± 0.01^aA^	4282.44 ± 0.01^aA^	27.61 ± 0.01^aA^	0.61 ± 0.01^aA^
	21	6.70 ± 0.01^aA^	11.50 ± 0.01^aA^	0.13 ± 0.01^bB^	3618.33 ± 0.01^bD^	21.13 ± 0.01^bD^	0.61 ± 0.01^aA^
CSMF4	1	6.58 ± 0.01^bA^	11.43 ± 0.01^bAB^	0.17 ± 0.01^aA^	3766.15 ± 0.01^aCD^	16.00 ± 0.01^aF^	0.23 ± 0.01^aC^
	21	6.70 ± 0.01^aA^	11.53 ± 0.01^aA^	0.12 ± 0.01^bB^	3137.26 ± 0.01^bG^	15.45 ± 0.01^aG^	0.23 ± 0.01^aC^
SM1	1	6.54 ± 0.01^bA^	11.47 ± 0.01^aA^	0.18 ± 0.01^aA^	3799.85 ± 0.01^aCD^	16.74 ± 0.01^aF^	0.15 ± 0.01^aD^
	21	6.65 ± 0.01^aA^	11.50 ± 0.01^aA^	0.12 ± 0.01^bB^	3198.74 ± 0.01^bG^	14.36 ± 0.01^bI^	0.15 ± 0.01^aD^
SM2	1	6.54 ± 0.01^bA^	11.43 ± 0.01^aAB^	0.18 ± 0.01^aA^	3776.15 ± 0.01^aCD^	14.58 ± 0.01^aI^	0.15 ± 0.01^aD^
	21	6.65 ± 0.01^aA^	11.50 ± 0.01^aA^	0.12 ± 0.01^bB^	3165.04 ± 0.01^bG^	13.13 ± 0.01^bJ^	0.15 ± 0.01^a^
SM3	1	6.59 ± 0.01^bA^	11.30 ± 0.01^bB^	0.18 ± 0.01^aA^	3731.70 ± 0.01^aCD^	12.66 ± 0.01^aK^	0.15 ± 0.01^aD^
	21	6.71 ± 0.01^aA^	11.53 ± 0.01^aA^	0.12 ± 0.01^bB^	3114.30 ± 0.01^bG^	11.99 ± 0.01^bL^	0.15 ± 0.01^aD^

Data are means ± standard error. Values with different letters are significantly different *p* < 0.05. Small letters in the column indicate the differences within the storage time. Capital letters in the column indicate the difference between the samples. CSMF1: semi-skimmed milk + 1% cocoa + 2% glucose; CSMF2: semi-skimmed milk + 2% cocoa + 2% glucose; CSMF3: semi-skimmed milk + 3% cocoa + 2% glucose; CSMF4: semi-skimmed milk + 2% glucose; SM1: semi-skimmed milk + 1% sterile distilled water + 2% glucose; SM2: semi-skimmed milk + 2% sterile distilled water + 2% glucose; SM3: semi-skimmed milk + 3% sterile distilled water + 2% glucose. AOX, antioxidant capacity (equivalent μmol Trolox/L); TPC, total polyphenol content (equivalent milligrams of gallic acid (GAE)/L); AAC, ascorbic acid (equivalent mg acid ascorbic/L).

### Design of PBs, viability of bacteria, and technological characteristics at 21-day storage

**FIGURE 1:**
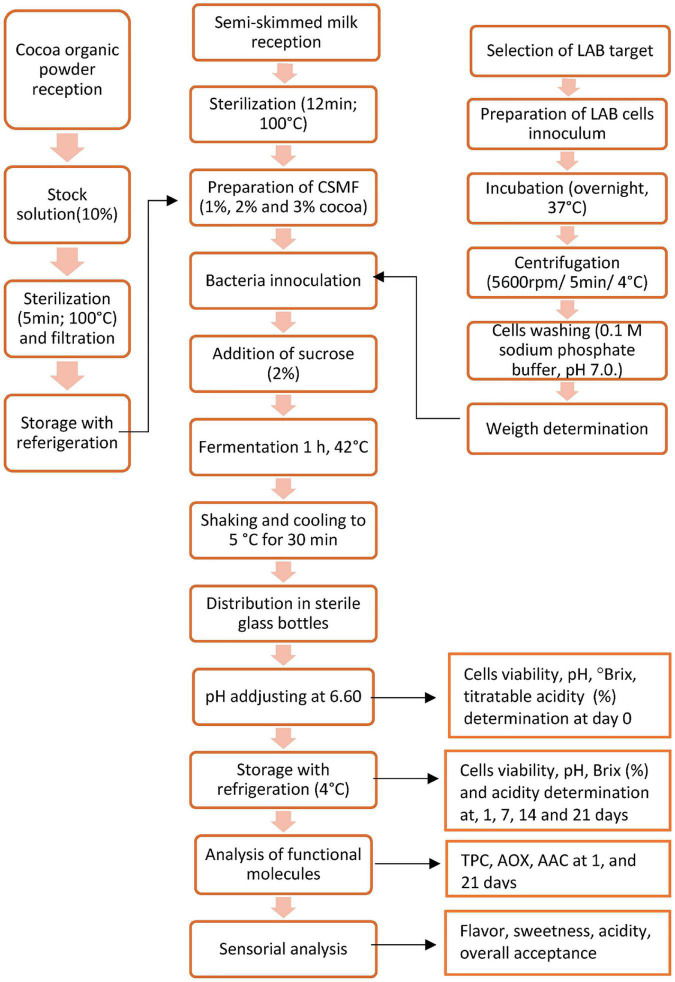
Workflow of the manufacturing of cocoa-based semi-skimmed milk beverage supplied with LAB strains. Legend: LAB, lactic acid bacteria; CSMF, semi-skimmed milk + 1–3% cocoa + 2% glucose; TPC, total polyphenol content (equivalent milligrams of gallic acid (GAE)/L); AOX, antioxidant capacity (equivalent μmol Trolox/L); AAC, ascorbic acid (equivalent mg acid ascorbic/L).

**FIGURE 2:**
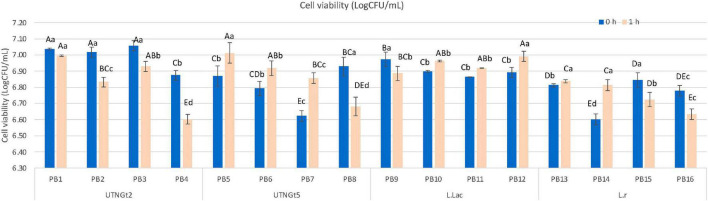
Cell viability (logCFU/ml) comparison of PBs beverages obtained without fermentation (0 h) and fermentation step (1 h). Bars are the means ± standard error. Values with different letters are significantly different *p* < 0.05. Capital letters show the differences within the method (Duncan’s test). Small letters show the differences within the formulation-with and without cocoa (LSD with Bonferroni correction). Legend: PB1–PB4: (CSMF1–CSMF4) + UTNGt2; PB5–PB8: (CSMF1–CSMF4) + UTNGt5; PB9–PB12: (CSMF1–CSMF4) + L.Lac; PB13–PB16: (CSMF1–CSMF4) + L.r. CSMF1: semi-skimmed milk + 1% cocoa + 2% glucose; CSMF2: semi-skimmed milk + 2% cocoa + 2% glucose; CSMF3: semi-skimmed milk + 3% cocoa + 2% glucose; CSMF4: semi-skimmed milk + 2% glucose.

Figure 1 shows the manufacturing flow of the PBs. Cell viability, appearance (liquid before viscosity), and overall impression at the end of storage were the critical criteria for the procedure selection. The pH value registered for each of the PB obtained with and without a fermentation step are shown in Supplementary Table 1. The results indicated that the pH varies with the method applied, the percentage of cocoa in the formulation, and the bacterial strain, therefore for optimization purpose, the pH was adjusted to 6.60 before storage. An adequate number of live probiotics (10^6^–10^7^ CFU/ml per grams of food) are recommended at the time of consumption to exhibit health benefits (27). Figure 2 showed the viability of LAB strains in the PBs registered on day 21 of storage with refrigeration. The results indicated that PBs obtained by the 2 procedures maintained the viability of the four-probiotic tested, nonetheless the final product obtained without the fermentation step showed changes in the appearance (yogurt formation) in the samples containing UTNGt5, L.Lac, and L.r by day 21 of storage. In addition, these products have a very low acceptance as the panelists perceived a bitter, acidic, and not pleasant taste (data not shown). Thus, the PBs obtained without fermentation step were considered not suitable for the obtention of a beverage supplied with LAB strains. The number of cell counts was above the threshold (<10^6^CFU/ml) in all beverages. A significant decrease (*p* < 0.05) in the cell population was recorded for PB4, PB8, PB12, and PB16 corresponding to cocoa-free beverages. This result suggested the positive effect of cocoa on bacterial survival. Complementary growth curve in media containing 5% cocoa indicated an increase in native LAB cell viability with the increasing of cocoa concentration (data not shown). The LAB strains tend to adhere to the cocoa particles and they sediment on the lower side of the assay tube as well as in the MRS media without cocoa. Nonetheless, the results indicated that the cell viability was related to the matrix (% of cocoa supplied), strain adaptability, and the method applied. Recent research suggests that microencapsulation of probiotics on liquid or dry vegetable-based matrices could maintain their viability throughout shelf-life (44). The beverages obtained with the native strains, UTNGt2 and UTNGt5, revealed a statistically significant difference (*p* < 0.05) in cell counts in the final PB product, with UTNGt2 showing high viability upon fortification and direct storage with refrigeration (0 h), and UTNGt5 showing high viability upon the fermentation step (1 h). Likewise, both reference strains have different behavior depending on the method, with L.Lac being more stable than L.r during storage for the fermentation step. Although the fermentation period was 1 h, this was a crucial step in maintaining the liquid state of the product throughout storage. Complementary studies with larger fermentation time (3 h) showed a drastic decrease in cell count and an increase in acidity along with gel formation in the final product (data not shown). The overall impression (%) was superior for PBs obtained with the fermentation step. Therefore, the procedure with the fermentation step (1 h) was chosen for the production and validation of the beverage. At the end of storage, more than 6.60 log CFU/ml of probiotics survived in all PBs, indicating the adaptability of the probiotic strain to the matrix. Thus, these formulations might exert prebiotic function allowing the LAB strains to survive or maintain throughout the storage with superior amount than the threshold value considered for a probiotic strain (45).

### Survival of lactic acid bacteria strains throughout storage on PBs obtained from the fermentation step method

**FIGURE 3:**
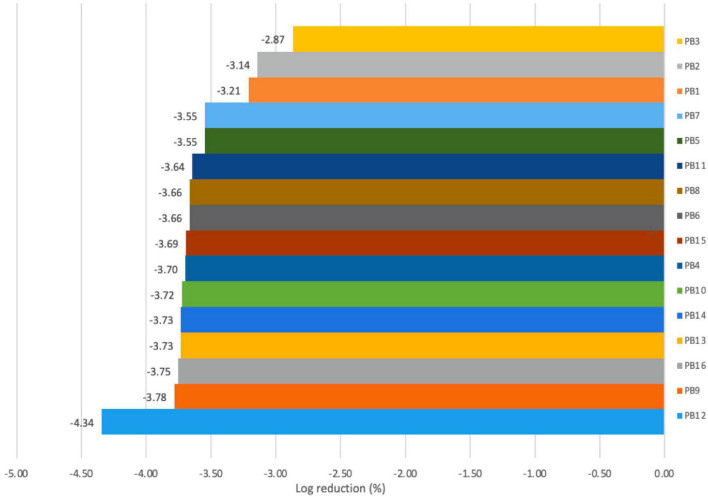
Log reduction (%) in the cell counts in PBs at the end of storage (day 21) obtained with fermentation step. Legend: PB1–PB4: (CSMF1–CSMF4) + UTNGt2; PB5–PB8: (CSMF1–CSMF4) + UTNGt5; PB9–PB12: (CSMF1–CSMF4) + L.Lac; PB13–PB16: (CSMF1–CSMF4) + L.r. CSMF1: semi-skimmed milk + 1% cocoa + 2% glucose; CSMF2: semi-skimmed milk + 2% cocoa + 2% glucose; CSMF3: semi-skimmed milk + 3% cocoa + 2% glucose; CSMF4: semi-skimmed milk + 2% glucose.

From a technological point of view, preserving cell viability during storage in new beverages supplemented with probiotic cultures is not a simple task, as viability can be affected by several factors, such as acid accumulation, lineage change, and interaction of the probiotic strain with the food matrix (46). In the present study, changes in viable cell counts (CFU/ml) of the four LAB strains in the final PBs obtained from the fermentation step were monitored during cold storage (4°C) for 21 days (Supplementary Figures 2A–D). Although a significant difference in viability (*p* < 0.05) between the combinations was observed, at the end of storage, all PB products contained more than 1 × 10^7^CFU/ml per portion (100 ml). This value was superior to the minimum count of probiotic bacteria intake recommended for probiotic strains (45). For the beverages obtained with UTNG2, the lowest cell reduction (3.7%) was observed in the cocoa-free sample (PB4), while in the cocoa-containing samples (PB1, PB2, PB3) the cell viability was slightly altered (2.87–3.21% log reduction), indicating that cocoa might impaired the viability loss during cold storage (Figure 3). In addition, the beverages obtained with UTNGt5 showed comparable cell viability reduction (3.55–3.66%) during storage. At day 21, the highest log reduction was registered for PB12 (4.34%) followed by PB9 (3.78%), corresponding to beverages containing L.r and L.Lac, strains, respectively (Figure 3). Although both reference strains (L.Lac and L.r) originated from breastmilk (47, 48), the adaptability to the newly matrices decreased compared with the native strains. Prior reports have shown that the milk chocolate matrix carrying the new probiotics *L. acidophilus* LDMB-01 showed an optimal probiotic viability throughout cold storage and a higher decrease in samples stored at 25°C (49). Similarly, another investigation revealed that the formulation of a milk chocolate matrix with encapsulated probiotics (*Lacticasei. casei* NCDC 298), sodium alginate (4%), and maize starch (2%) was a protective matrix to maintain the viability of probiotics (50). In addition, a semi-sweet chocolate supplemented with freeze-dried probiotics (*L. acidophilus* LA3 and *B. animalis* subsp. *lactis* BLC1) maintained the bacterial viability during chocolate manufacturing (10). In the present study, the native strains had better cell viability than the reference strains in the final beverages. We have observed that the bacterial cells and cocoa particles adhere to the bottom of the glass bottles. However, the good survival in cocoa-supplemented semi-skimmed milk compared to the cocoa-free beverages containing each individual target bacteria is credited to the cocoa-semi skimmed milk matrix as a protector of LAB cells which might help preserve the viability throughout gastrointestinal transit. We speculated that such protective effects could be attributed to the high polyphenols content of native cocoa “aroma fina” (19), as well as the habituation strength of native LABs, which originated from cocoa, and their survival rate in the target CSMF matrix, but further research is needed to prove this statement.

### Physicochemical properties of PBs during storage with refrigeration

**FIGURE 4:**
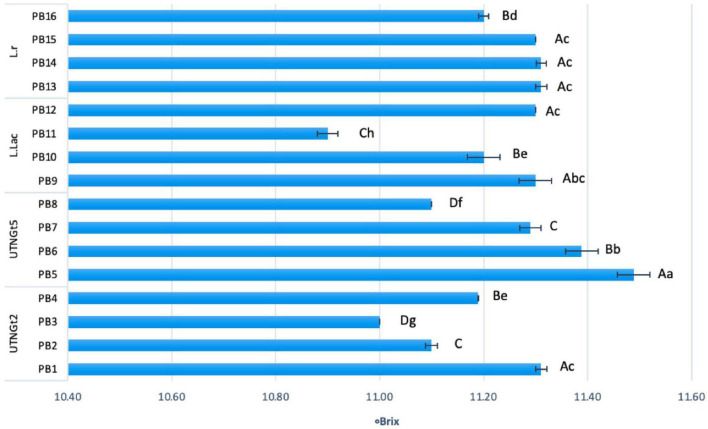
Total solids (°Brix) comparison between the PBs at day 21 of storage. Bars are the means ± standard error. Values with different letters are significantly different *p* < 0.05. Small letters show the difference between the beverages (LSD with Bonferroni correction); Capital letters indicate the differences within strain (Duncan’s test). Legend: PB1–PB4: (CSMF1–CSMF4) + UTNGt2; PB5–PB8: (CSMF1–CSMF4) + UTNGt5; PB9–PB12: (CSMF1–CSMF4) + L.Lac; PB13–PB16: (CSMF1–CSMF4) + L.r. CSMF1: semi-skimmed milk + 1% cocoa + 2% glucose; CSMF2: semi-skimmed milk + 2% cocoa + 2% glucose; CSMF3: semi-skimmed milk + 3% cocoa + 2% glucose; CSMF4: semi-skimmed milk + 2% glucose.

The changes of pH and acidity throughout the storage is illustrated in Supplementary Figures 3A–D. The lowest acidity was recorded for beverages containing L.Lac (0.25–0.29) and UTNGt5 (0.30–0.36), followed by L.r (0.43–0.48) and UTNGt2 (0.42–0.50) supplemented matrices. Nonetheless, the acidity did not change the viscosity of the final product, which showed liquid consistency throughout the storage. Moreover, during storage a gradual decrease in total solids was detected in all beverages (data not shown). Based on the results, we appreciate that the variation in total solids was related to the LAB strains used and their ability to metabolize sucrose in the matrix. At day 21 of storage, statistically significant differences (*p* < 0.05) between beverages were observed (Figure 4). The lowest percentage of total solids was recorded for samples with 3% cocoa and probiotic UTNGt2 and L.Lac strains (PB3 and PB11), while for the samples obtained with UTNGt5 and L.r, the lowest percentage of total solids was recorded for cocoa-free beverages (PB8 and PB16). The highest total solids content was determined in PB5 and PB6 obtained with UTNGt5, suggesting that the UTNGt5 might retain the sugar content instead of metabolizing it. This result coincides with the preservation of cell viability where the smallest decreases in cell counts were recorded for the beverages obtained with UTNGt5. Recent studies have indicated that total solids, fats, and proteins generate a protective matrix for probiotic cells (3). Besides, the L.r strain showed similar behavior to UTNGt5, for which no significant difference (*p* > 0.05) was observed in total solids. Although viscosity was not determined, beverages made with the four probiotic strains remained liquid throughout refrigeration. A slight change was observed in the samples containing reference L.r and 3% cocoa, showing little thickness at day 21. Based on this study, we concluded that the beverages obtained with each of the strains tested altered at the lesser extent the physicochemical properties of the semi-skimmed milk cocoa matrix, these changes might be attributed to the specie post-acidification capacity and the matrix composition.

### The effect of lactic acid bacteria strains on the total protein and fat content

**FIGURE 5:**
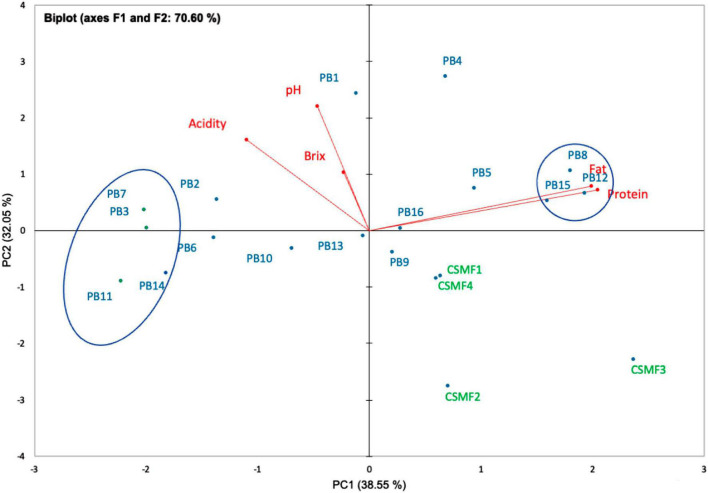
Biplot PCA analysis of five variables (pH, titratable acidity, °Brix, total protein content and fat) of PBs and CSMFs at day 21 of storage; the rectangles marked the close-related beverages. Legend: PB1–PB4: (CSMF1–CSMF4) + UTNGt2; PB5–PB8: (CSMF1–CSMF4) + UTNGt5; PB9–PB12: (CSMF1–CSMF4) + L.Lac; PB13–PB16: (CSMF1–CSMF4) + L.r. CSMF1: semi-skimmed milk + 1% cocoa + 2% glucose; CSMF2: semi-skimmed milk + 2% cocoa + 2% glucose; CSMF3: semi-skimmed milk + 3% cocoa + 2% glucose; CSMF4: semi-skimmed milk + 2% glucose.

Proteins are relevant biologically active resources of food, however, the potential biological activity of cocoa oligopeptides in semi-skimmed milk matrix and in relation to probiotic cells has not been sufficiently studied. Based on the results, there were differences between the matrices in terms of protein and fat content (%), which varies with the composition of the matrices (% cocoa) and the target supplemented bacterial strain. The protein and fat content of the cocoa semi-skimmed milk (CSMF) matrices used in this study are shown in Supplementary Table 3. As a reference, the established limits of the Ecuadorian standard for semi-skimmed milk [(37): NTE INEN 701:2009], fermented milk [(25): FAO CXS-243:2003], and cocoa powder [(38): NTE INEN 620:2017] were considered. The results suggest that the bacterial strain in association with the matrices (cocoa-free and cocoa-supplied matrices) may vary the total protein and fat content to a lesser extent. During fermentation, protein and fat modifications cause corresponding changes in taste, aroma, texture, and nutritional value of the product (51). Furthermore, PCA analysis of 5 variables (pH, acidity, °Brix, protein, and fat) on day 21 showed a separation between the CSMFs and PBs. The F1 component explained 38.55% of the total variance being loaded in the positive (+) direction with protein and fat, while the F2 explained 32.05% of the variance being loaded in the negative (–) direction, with °Brix, acidity, and pH (Figure 5). The protein and fat vectors are those that most influence the main component or axis F1 (43.43 and 41.50%), while the pH and acidity vectors contribute with 50.41 and 26.74%, respectively to the main component F2. In addition, the vector °Brix influenced with greater superiority on the main component F3 (77.72%). We observed that the variables protein and fat were close together, indicating a high correlation between these cutoff values throughout the experiment. Early research showed that protein variation is related to the breakdown of milk protein during the fermentation process (52). However, the results indicated that the high cocoa concentration (3%) and the supplied LAB species altered to a lesser extent the total protein and fat content and coincide with the diminution of the pH and acidity, as noted with the PB3, PB7, PB11, and PB14, as they were located distant from this vector. Further complementary analyses are required to determine the nature of these changes. During storage, within each PB, there were no statistically significant differences (*p* > 0.05) in the content of protein in fat (data not shown). It seems that the storage does not cause any change in the final matrices suggesting its stability. In addition, these levels in each of the beverages remain above the CODEX Alimentarius [(25): FAO CXS-243:2003] recommended limit of 2.7% for fermented milk (flavored) beverages.

### The effect of lactic acid bacteria strains on total polyphenolic compounds, antioxidant capacity, and ascorbic acid content in PBs during storage

**FIGURE 6:**
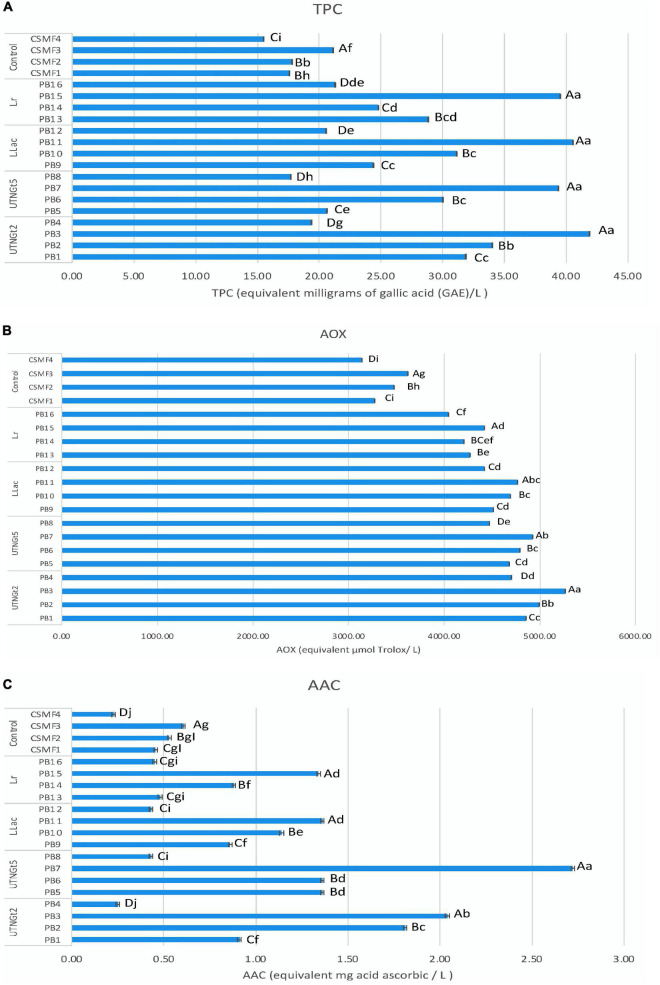
Effect of bacteria on TPC **(A)**, AOX **(B)**, and AAC **(C)** at the end of storage (21 day). Bars are the means ± standard error. Values with different letters are significantly different *p* < 0.05. Small letters show the difference between the beverages (LSD with Bonferroni correction); Capital letter show the differences within the beverage group (Duncan’s test). Legend: PB1–PB4: (CSMF1–CSMF4) + UTNGt2; PB5–PB8: (CSMF1–CSMF4) + UTNGt5; PB9–PB12: (CSMF1–CSMF4) + L.Lac; PB13–PB16: (CSMF1–CSMF4) + L.r. CSMF1: semi-skimmed milk + 1% cocoa + 2% glucose; CSMF2: semi-skimmed milk + 2% cocoa + 2% glucose; CSMF3: semi-skimmed milk + 3% cocoa + 2% glucose; CSMF4: semi-skimmed milk + 2% glucose. AOX, antioxidant capacity (equivalent μmol Trolox/L); TPC, total polyphenol content [equivalent milligrams of gallic acid (GAE)/L]; AAC, ascorbic acid (equivalent mg acid ascorbic/L).

**FIGURE 7:**
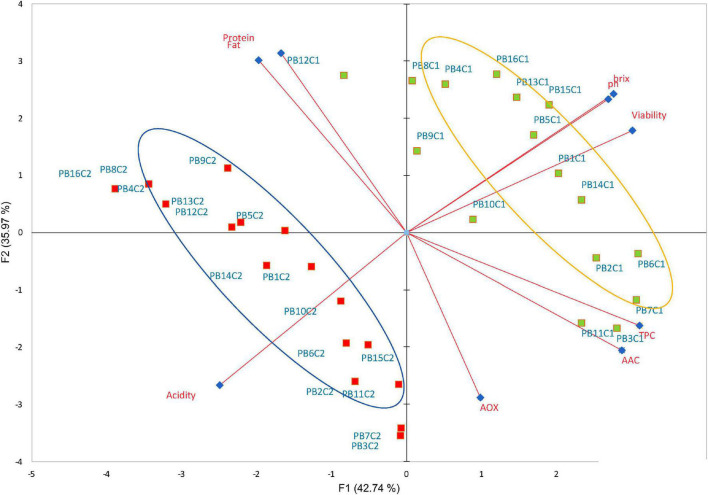
Biplot PCA analysis of the nine variables (pH, titratable acidity, Brix, TPC, AOX, AAC, protein, and fat) of PBs obtained with the four probiotic strains. The green and red rectangles represent the PBs at day 1 and 21, respectively. Legend: PB1–PB4: (CSMF1–CSMF4) + UTNGt2; PB5–PB8: (CSMF1–CSMF4) + UTNGt5; PB9–PB12: (CSMF1–CSMF4) + L.Lac; PB13–PB16: (CSMF1–CSMF4) + L.r. CSMF1: semi-skimmed milk + 1% cocoa + 2% glucose; CSMF2: semi-skimmed milk + 2% cocoa + 2% glucose; CSMF3: semi-skimmed milk + 3% cocoa + 2% glucose; CSMF4: semi-skimmed milk + 2% glucose; C1–C2: values registered at 1 and 21 days for each variable. TPC, total polyphenol content; AOX, antioxidant activity; AAC, acid ascorbic content.

Cocoa polyphenols impart bitterness and astringency and contribute to the green and fruity flavor of the beans (53), in addition to acting as prebiotic mechanisms by modulating microbial diversity, promoting the proliferation of various bacteria, and suppressing potentially pathogenic ones (54). Likewise, milk chocolate is known to be a source of diverse active compounds with essential antioxidant activity (55, 56). Peptides from the digestion of milk proteins are said to have an antioxidant impact (57), while cocoa and its products are rich in polyphenols such as flavanols (58). These compounds exert antioxidant and anti-inflammatory effects and have the potential to be used as probiotic carriers in dairy products leading resulting in improved bowel cleansing (9). In this study, the data obtained indicated that both cocoa and LAB strains have an impact on the overall functional molecule concentration in the final beverages supplemented with bacteria at the final day (21) of storage. Within the group, statistically significant differences (*p* < 0.05) in TPC, AOX, and ACC were observed with the highest content detected in the CSMF3 (3% cocoa) supplemented with bacteria (Figures 6A–C). The results showed that the TPC varies with the presence of certain LAB cultivars and the cocoa concentration in the matrix (Figure 6A). Non-cocoa beverages made with native strains showed the lowest TPC values comparable with the CSMFs. Recent research indicates that the probiotic strains supplemented cashew milk-based yogurt showed an increment in the total phenolic content, antioxidant capacity, and flavonoid content, this increase being subjectively associated with the probiotic species (59). Likewise, the AOX activity profile varies with the LAB species and cocoa concentration, with superior values registered for the beverages containing the native UTNGt2 and UTNGt5 strains followed by L.Lac, and L.r in the matrices containing 3% cocoa (Figure 6B). A recent review article found that the use of probiotic strains significantly optimizes the antioxidant status of dairy products (60). The highest AAC content was determined in the PB3 and PB7 samples corresponding to the CSMF3 matrices containing 3% cocoa and the UTNGt2 and UTNGt5 strains (Figure 6C). Given the results, we speculate that this increase in the AAC of beverages containing the native strains may have something to do with their origin (cocoa), a claim that should be further investigated. Vitamin C is one of the best-known antioxidants that play a role in protecting body cells from oxidative stress (61). Its degradation during storage has been demonstrated in various products (62). From the genome sequencing annotation, we showed that the UTNGt2 strain harbor genes encoding the biosynthesis of vitamin B9 (7 genes) and riboflavin (6 genes) (21). Besides, the antimicrobial strength of UTNGt2 against various pathogens was demonstrated, suggesting its advantage for use in foods for greater protection against harmful microorganisms. Thus, the use of probiotics as alternative to expensive chemical production of vitamins is of interest, several research are conducted to elucidate the biosynthetic pathway of these vitamins (63). Although a slight diminution during storage was observed, the final products contain more AAC than the beverages free of LAB strains. Moreover, to evaluate the effect of bacteria on the final beverages, a comparison between the variables (8) between the PBs and their matrix counterpart (CSMFs) was performed at the end of storage. A biplot was constructed using the PCA scores and factor loading to equate the similarities of the variables obtained (Supplementary Figure 4). The variable F1 explained 51.24% of the total variance, while F2 explained 21.08%. Based on the results, the PB4, PB8, PB12 and PB15, were characterized by grater fat and protein, while PB2, PB3, and PB7, corresponding to beverages obtained with the native LAB strains were characterized by high TPC, AOX, and AAC (Supplementary Figure 4). Overall, our analysis showed that adding specific LAB strains to distinct cocoa-based semi-skimmed milk matrices significantly increases TPC, AOX, and AAC status, so consuming these products could improve the antioxidant capacity of the organism through retention against oxidative stress and damage. Further studies are needed to prove this statement. Furthermore, we compared nine variables (viability, pH, °Brix, acidity, AOX, TPC, AAC, protein, and fat) of the PBs at the initial and final day of storage. PCA of the 9 factors demonstrated a clear separation between the beverages on days 1 and 21 (Figure 7). The variable F1 explained 42.74% of the total variance, while F2 explained 35.97%. The results showed that the beverages at day 1 of storage were characterized by greater pH, °Brix, viability, TPC, and AAC, while at day 21 had greater levels of protein, fat, and AOX. The contribution of the variables was 17.25% TPC, 16.26% viability, 14.84% AAC, 13.7% °Brix, 12.99% pH, and 11.13% acidity for F1, and 17.68% protein and 16.29% fat for F2. We observed that the variables (°Brix and pH) and (protein and fat) were close together, indicating a high correlation between these cutoff values throughout the experiment. In addition, the AOX vector forms an angle of about 90° between the other vectors, meaning that is an independent vector, or it has a low relation with the other variables. However, a Pearson correlation coefficient test was performed to study in detail the intensity of the correlations between the variables (Table 2). The greatest correlation was seen in the protein and fat variables with a value of 0.918, followed by TPC and AAC with a value of 0.814, with decreases in protein and fat and increased cell viability. In addition, the highest correlation occurs between cell viability and pH, followed by cell viability and acidity, with a value of 0.783 and –0.747, respectively. As the cell viability increased, the acidity decreased. Finally, the variables pH and °Brix, were highly correlated with a value of 0.744, with the increasing of cell counts. Overall, the supplementation of cocoa based semi-skimmed milk matrices with native LAB strains originated from wild cocoa, results in increased natural polyphenols, vitamin C, and antioxidant capacity, these promising results might help to further develop strategies to obtain novel functional fermented foods with enhanced benefits.

**TABLE 2:** Pearson correlation coefficient to the nine variables.

Variables	AOX	TPC	AAC	Protein	Fat	pH	°Brix	Acidity	Viability
AOX	**1**	**0.411**	**0.585**	–0.344	**−−0.388**	–0.235	–0.276	0.131	–0.145
TPC	**0.411**	**1**	**0.814**	**−−0.603**	**−−0.667**	0.331	0.233	–0.250	**0.361**
AAC	**0.585**	**0.814**	**1**	**−−0.450**	**−−0.511**	0.261	0.268	–0.265	0.307
Protein	–0.344	**−−0.603**	**−−0.450**	**1**	**0.918**	0.037	0.149	–0.058	0.089
Fat	**−−0.388**	**−−0.667**	**−−0.511**	**0.918**	**1**	0.007	0.093	–0.040	–0.001
pH	–0.235	0.331	0.261	0.037	0.007	**1**	**0.744**	**−−0.790**	**0.783**
°Brix	–0.276	0.233	0.268	0.149	0.093	**0.744**	**1**	**−−0.755**	**0.796**
Acidity	0.131	–0.250	–0.265	–0.058	–0.040	**−−0.790**	**−−0.755**	**1**	**−−0.747**
Viability	–0.145	**0.361**	0.307	0.089	–0.001	**0.783**	**0.796**	**−−0.747**	**1**

Values in bold are different from 0 with a significance level alpha = 0.05. AOX, antioxidant capacity (equivalent μmol Trolox/L); TPC, total polyphenol content (equivalent milligrams of gallic acid (GAE)/L); AAC, ascorbic acid (equivalent mg acid ascorbic/L).

### Overall sensory impression of PBs obtained from the fermentation step

**FIGURE 8:**
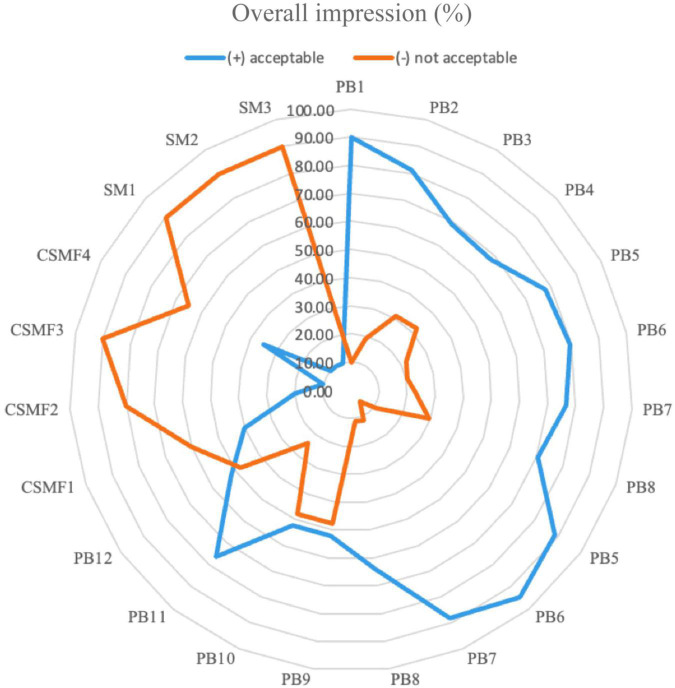
Radar plot of the overall acceptance of the PBs compared with CSMFs and SMs at day 21. Legend: PB1–PB4: (CSMF1–CSMF4) + UTNGt2; PB5–PB8: (CSMF1–CSMF4) + UTNGt5; PB9–PB12: (CSMF1–CSMF4) + L.Lac; PB13–PB16: (CSMF1–CSMF4) + L.r. CSMF1: semi-skimmed milk + 1% cocoa + 2% glucose; CSMF2: semi-skimmed milk + 2% cocoa + 2% glucose; CSMF3: semi-skimmed milk + 3% cocoa + 2% glucose; CSMF4: semi-skimmed milk + 2% glucose; SM1: semi-skimmed milk + 1% sterile distilled water + 2% glucose; SM2: semi-skimmed milk + 2% sterile distilled water + 2% glucose; SM3: semi-skimmed milk + 3% sterile distilled water + 2% glucose.

Sensory examinations were evaluated throughout the storage in the PBs, CSMFs, and SMs, respectively. The overall acceptance is shown in Figure 8. The results showed that the addition of the bacterial strains to the CSMF has an impact on the sensory properties of the final beverages, with the PBs obtained with the UTNGt2, UTNGt5, and L.Lac strains and 1–2% cocoa being better accepted by the panelists (>78%) compared to the matrix containing L.r strain. At day 21, CSMF and SM were poorly accepted (<40%) by the test subjects as they were found off-flavor compared to the beverages supplied with bacteria. It appears that adding bacteria to CSMF preserves flavor in the final product. The results showed that each of the beverages was liquid, had a cocoa flavor except for the non-cocoa samples, and had weak acidity. In terms of sweetness, more than 40% of the beverages were weak (9%), and moderately sweet (47–48%). In addition, 1 and 2% supplied cocoa and cocoa-free beverages obtained with the UTNGt5 strain were perceived as quite sweet compared to the other beverages (data not shown). This coincides with a higher proportion of total solids detected in the PB5 and PB6 samples (Figure 4). Among all strains tested, the PB containing *L. reuteri* were less accepted. Although perceived as bitter, 76% of the participants preferred the PB15, which contained L.r and 3% cocoa. This beverage showed high levels of TPC, less protein and less fat than its counterpart without cocoa (PB16), which could explain the bitter taste. Early research indicates that cocoa polyphenols and alkaloids confer bitterness and astringency, being involved in the palatability of foods (53). In addition, supplementation with the probiotic *Bacillus indicus* HU36 had no deleterious impact on customer acceptance of probiotic milk chocolate (64). Similarly, when the chocolates were enriched with probiotics, *Lacticasei. casei*, and *Lacticasei. paracasei* powders, they turned out to be like those of the non-inoculated samples (65). Additionally, some studies have shown that adding probiotics to certain foods has minimal or no sensory effects (66). Besides, the flavor might be related to the proportion of theobromine in the final product, however, more studies are needed to confirm this statement. Previous research indicated that the concentration of this alkaloid is related to the cocoa variety, Criollo cocoa being less bitter due to the higher caffeine content. Nonetheless, a substantially lower theobromine content results in the perception of a consumer up to eleven times more bitter than caffeine itself (67). Finally, at the end of storage, the beverages were free from harmful bacteria (no coliforms, *Salmonella* spp., *E. coli* detected), yeasts, and molds, indicating that they were safe for consumption.

Taken together, if cell viability coupled with enhanced functional molecules (TPC, AOX, and AAC) are considered as the main selection criteria, the PBs (16) would be classified as entirely new drinks supplemented with native LAB strain with probiotic-like features, regardless of the effect of cocoa concentration and the strain provided. Considering protein and fat coupled with the sensorial analysis, beverages obtained with both native strains (UTNGt2 and UTNGt5) supplying semi-skimmed milk containing 1% and 2% cocoa have the highest acceptance according to the panelist’s selection. Likewise, beverages obtained with the reference L.Lac, using the matrices containing 1% and 2% cocoa were better appreciated than the reference L.r, where the beverage PB15 only (containing 3% cocoa) received better acceptance. Thus, for this study, the addition of native strains to CSMFs results in good acceptance when comparing to the reference strains.

## Conclusion

In this study, the fortification of different cocoa semi-skimmed milk matrices with native LAB strains with probiotic potential through an established method with 1 h of fermentation, results in the production of novel beverages with acceptable physicochemical characteristics and organoleptic qualities. Based on this research, the 1% and 2% cocoa semi-skimmed milk formulations were effective carriers of native LAB strains showing acceptable viability throughout cold storage. The addition of two native LAB strains alters the physicochemical quality of cocoa semi-skimmed milk formulations to a lesser extent, in which the bioactive molecules (TPC, AOX, and AAC) have improved significantly (*p* < 0.05) with increasing cocoa concentration and depending on the strain used. Considering the total AAC content, the beverages obtained with the native (UTNGt2 and UTNGt5) were better than the reference (L.Lac and L.r) strains. However, the technology proposed here can be considered a suitable alternative to produce small and/or medium-scale cocoa-based beverages supplemented with native LAB strains; further research is required to assess the overall effects, safety, and toxicity of probiotics.

## Data availability statement

The original contributions presented in this study are included in the article/Supplementary material, further inquiries can be directed to the corresponding author.

## Ethics statement

The studies involving human participants were reviewed and approved by Comité de Bioética, Universidad Técnica del Norte. The patients/participants provided their written informed consent to participate in this study.

## Author contributions

GNT contributed to conceptualization, methodology, data curation, supervision, project administration, funding acquisition, and writing – review and editing. Both authors contributed to the formal investigation, analysis, and statistical analysis.
