# Supplementary material for: Fortification of cocoa semi-skimmed milk formulations with native lactic acid bacteria: Cell viability, physicochemical and functional properties for developing novel foods
Source: Front Nutr. 2022 Oct 13;9:1008871. doi: 10.3389/fnut.2022.1008871 (PMC9608143; doi:10.3389/fnut.2022.1008871)

**Supplementary Figure 1.** Changes (%) in AOX, TPC, and AAC at the end of storage (day 21). Legend: CSMF1: semi-skimmed milk + 1% cocoa + 2% glucose; CSMF2: semi-skimmed milk + 2% cocoa + 2% glucose; CSMF3: semi-skimmed milk + 3% cocoa + 2% glucose; CSMF4: semi-skimmed milk + 2% glucose; SM1: semi-skimmed milk + 1% sterile distilled water + 2% glucose; SM2: semi-skimmed milk + 2% sterile distilled water + 2% glucose; SM3: semi-skimmed milk + 3% sterile distilled water + 2% glucose; TPC: total polyphenol content (equivalent milligrams of gallic acid (GAE)/L); AOX: Antioxidant capacity (equivalent µmol Trolox/ L); AAC: ascorbic acid (equivalent mg acid ascorbic / L).


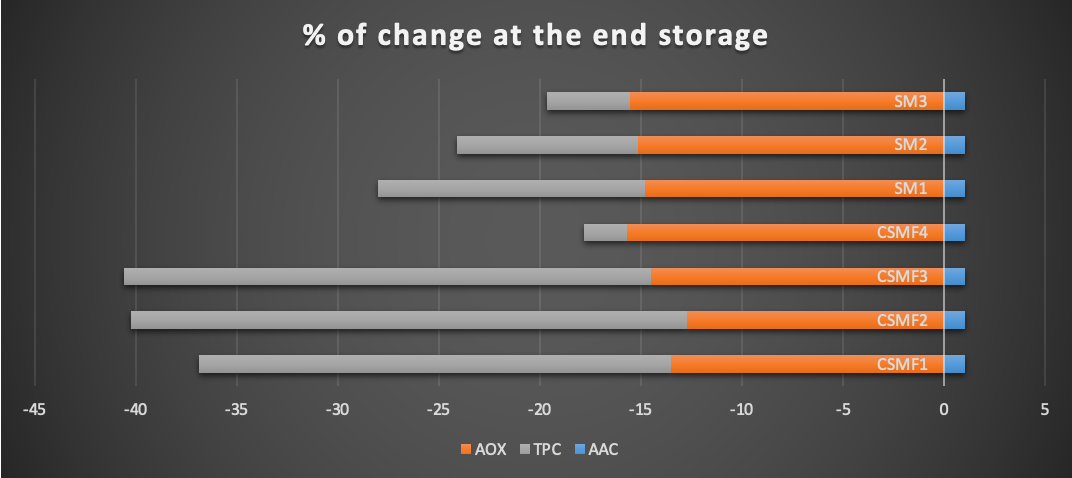


**Supplementary Figure 2**. Cell viability variation in PBs throughout storage with refrigeration. A). UTNGt2; B). UTNGt5; C). L.Lac; D) L.r. Bars are the means ± standard error. Values with different letters are significantly different *p* < 0.05. Capital letters indicate the differences within the storage time (Tukey test). Legend: PB1-PB4: (CSMF1-CSMF4) + UTNGt2; PB5-PB8: (CSMF1- CSMF4) + UTNGt5; PB9-PB12: (CSMF1-CSMF4) + L.Lac; PB13-PB16: (CSMF1-CSMF4) + L.r. CSMF1:semi-skimmed milk + 1% cocoa + 2% glucose; CSMF2: semi-skimmed milk + 2% cocoa + 2% glucose; CSMF3: semi-skimmed milk + 3% cocoa + 2% glucose; CSMF4: semi-skimmed milk + 2% glucose

A).


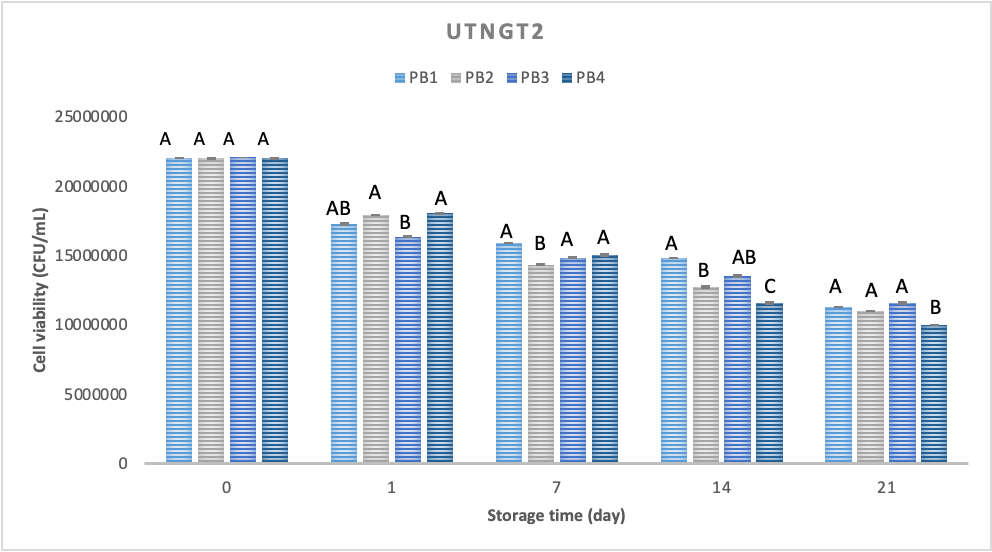


B).

C).

D).

**Supplementary Figure 3.** Variation of pH and titratable acidity (%) in PBs throughout storage with refrigeration. A). UTNGt2; B). UTNGt5; C). L.Lac; D) L.r . Legend: PB1-PB4: (CSMF1-CSMF4) + UTNGt2; PB5-PB8: (CSMF1- CSMF4) + UTNGt5; PB9-PB12: (CSMF1-CSMF4) + L.Lac; PB13-PB16: (CSMF1-CSMF4) + L.r. CSMF1:semi-skimmed milk + 1% cocoa + 2% glucose; CSMF2: semi-skimmed milk + 2% cocoa + 2% glucose; CSMF3: semi-skimmed milk + 3% cocoa + 2% glucose; CSMF4: semi-skimmed milk + 2% glucose.

A).


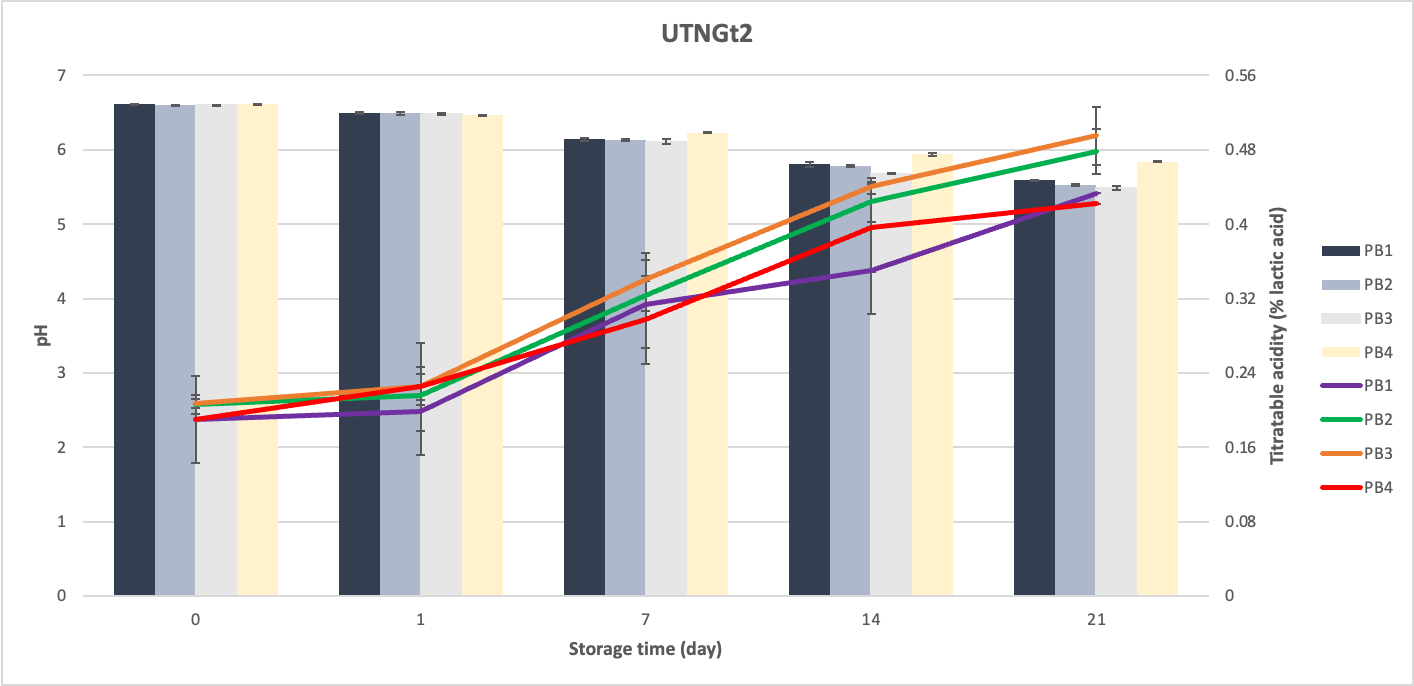


B.)

C).

D).

**Supplementary Figure 4.** Biplot PCA analysis of 8 variables (pH, titratable acidity, °Brix, protein, fat, TPC, AOX, and AAC) of PBs and CSMFs at day 21 of storage. The colored rectangles marked the close-related beverages. Legend: PB1-PB4: (CSMF1-CSMF4) + UTNGt2; PB5-PB8: (CSMF1- CSMF4) + UTNGt5; PB9-PB12: (CSMF1-CSMF4) + L.Lac; PB13-PB16: (CSMF1-CSMF4) + L.r. CSMF1:semi-skimmed milk + 1% cocoa + 2% glucose; CSMF2: semi-skimmed milk + 2% cocoa + 2% glucose; CSMF3: semi-skimmed milk + 3% cocoa + 2% glucose; CSMF4: semi-skimmed milk + 2% glucose. TPC: total polyphenol content (equivalent milligrams of gallic acid (GAE)/L); AOX: Antioxidant capacity (equivalent µmol Trolox/ L); AAC: ascorbic acid (equivalent mg acid ascorbic / L).


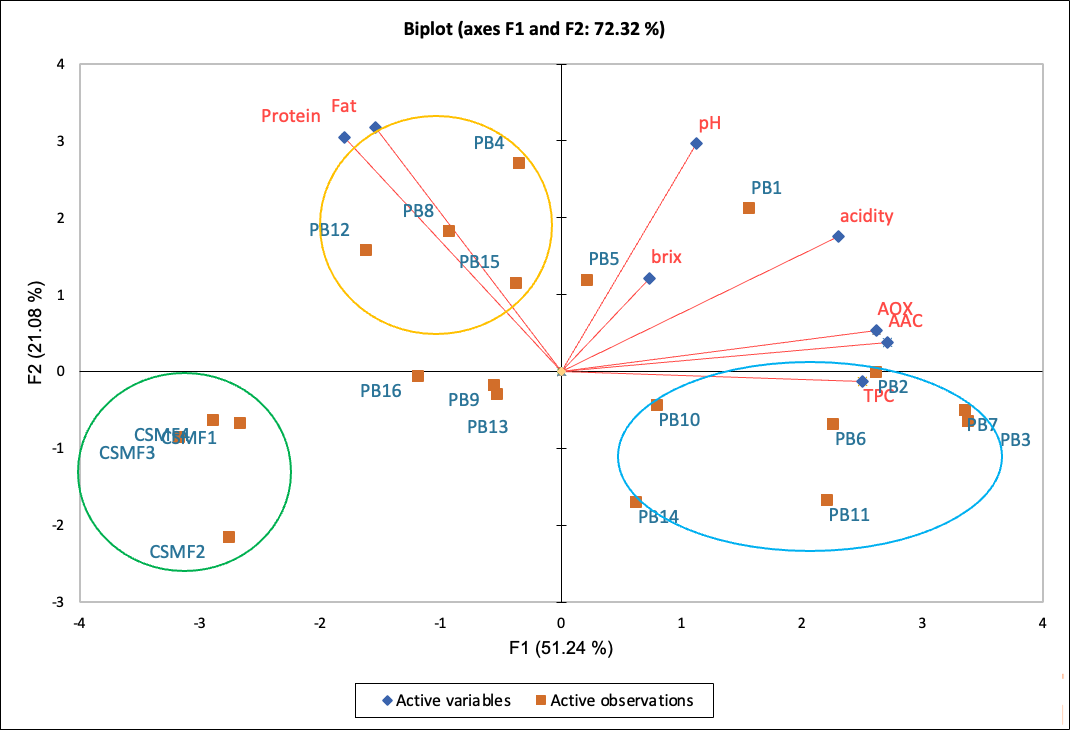

Supplement: Supplementary file 1 [file Data_Sheet_1.docx]
