# Supplementary material for: Fortification of cocoa semi-skimmed milk formulations with native lactic acid bacteria: Cell viability, physicochemical and functional properties for developing novel foods
Source: Front Nutr. 2022 Oct 13;9:1008871. doi: 10.3389/fnut.2022.1008871 (PMC9608143; doi:10.3389/fnut.2022.1008871)
Supplement: Supplementary file 2 [file Data_Sheet_2.docx]

**Supplementary Table 1.** Description of PBs and the determined pH in the final product obtained without (0 h) and with fermentation step (1h)

| **Beverage code** | **Bacteria** | **Matrix code** | **Matrix composition** | **No fermentation pH (0h)** | **With fermentation**  **pH (1h)** |
| --- | --- | --- | --- | --- | --- |
| PB1 | UTNGt2 | CSMF1 | semi-skimmed milk + 1% cocoa | 6.44 | 5.88 |
| PB2 |  | CSMF2 | semi-skimmed milk + 2% cocoa + 2% glucose | 6.42 | 5.85 |
| PB3 |  | CSMF3 | semi-skimmed milk + 3% cocoa + 2% glucose | 6.41 | 5.85 |
| PB4 |  | CSMF4 | semi-skimmed milk + 2% glucose (cocoa-free) | 6.52 | 5.93 |
| PB5 | UTNGt5 | CSMF1 | semi-skimmed milk + 1% cocoa | 6.45 | 6.05 |
| PB6 |  | CSMF2 | semi-skimmed milk + 2% cocoa + 2% glucose | 6.43 | 6.02 |
| PB7 |  | CSMF3 | semi-skimmed milk + 3% cocoa + 2% glucose | 6.44 | 5.99 |
| PB8 |  | CSMF4 | semi-skimmed milk + 2% glucose (cocoa-free) | 6.41 | 6.05 |
| PB9 | L.Lac | CSMF1 | semi-skimmed milk + 1% cocoa | 6.43 | 6.07 |
| PB10 |  | CSMF2 | semi-skimmed milk + 2% cocoa + 2% glucose | 6.40 | 6.02 |
| PB11 |  | CSMF3 | semi-skimmed milk + 3% cocoa + 2% glucose | 6.39 | 6.00 |
| PB12 |  | CSMF4 | semi-skimmed milk + 2% glucose (cocoa-free) | 6.49 | 6.07 |
| PB13 | L.r | CSMF1 | semi-skimmed milk + 1% cocoa | 6.43 | 6.11 |
| PB14 |  | CSMF2 | semi-skimmed milk + 2% cocoa + 2% glucose | 6.40 | 6.06 |
| PB15 |  | CSMF3 | semi-skimmed milk + 3% cocoa + 2% glucose | 6.38 | 6.03 |
| PB16 |  | CSMF4 | semi-skimmed milk + 2% glucose (cocoa-free) | 6.48 | 6.10 |

**Supplementary Table 2.** Raw materials physicochemical and functional characteristics.

| Samples | pH | Total solids  (° Brix) | Acidity* | AOX | TPC | AAC |
| --- | --- | --- | --- | --- | --- | --- |
| Sterile 1% cocoa solution | 5.08 ± 0.01^b^ | 4.50 ± 0.01^c^ | 0.06 ± 0.01^c^ | 4617.33 ± 0.01^c^ | 25.38 ± 0.01^g^ | 0.38 ± 0.01^d^ |
| Sterile 2% cocoa solution | 5.06 ± 0.01^b^ | 4.50 ± 0.01^c^ | 0.05 ± 0.01^c^ | 4742.15 ± 0.01^b^ | 41.61 ± 0.01^f^ | 1.97 ± 0.01^b^ |
| Sterile 3% cocoa solution | 5.04 ± 0.01^b^ | 4.50 ± 0.01^c^ | 0.04 ± 0.01^c^ | 5253.26 ± 0.01^a^ | 51.64 ± 0.01^e^ | 4.16 ± 0.01^a^ |
| UHT semi-skimmed milk | 6.66 ± 0.01^a^ | 10.90 ± 0.01^a^ | 0.14 ± 0.01^a^ | 4076.11 ± 0.01^f^ | 103.62 ± 0.01^c^ | 0.08 ± 0.01^f^ |
| Autoclaved semi-skimmed milk (12 min, 100°C) | 6.60 ± 0.01^a^ | 10.70 ± 0.01^a^ | 0.15 ± 0.01^a^ | 4394.30 ± 0.01^e^ | 110.07 ± 0.01^b^ | 0.15 ± 0.01^e^ |
| Whole milk UHT | 6.61 ± 0.01^a^ | 10.60 ± 0.01^a^ | 0.14 ± 0.01^a^ | 4658.37 ± 0.01^bc^ | 145.35 ± 0.01^a^ | 0.38 ± 0.01^d^ |
| Crude whole milk (no sterile) | 6.71 ± 0.01^a^ | 9.70 ± 0.01^b^ | 0.13 ± 0.01^b^ | 4533.56 ± 0.01^d^ | 98.80 ± 0.01^d^ | 0.53 ± 0.01^c^ |

Note: * % of citric acid in the cocoa samples; % of lactic acid in milk samples. Data are means ± standard error. Values with different letters in the column are significantly different *p* < 0.05. AOX: Antioxidant capacity (equivalent µmol Trolox/ L); TPC: total polyphenol content (equivalent milligrams of gallic acid (GAE)/L); AAC: ascorbic acid (equivalent mg acid ascorbic / L)

**Supplementary Table 3**. Registered values of protein and fat in CSMF matrices.

| **Samples** | **Total protein (mass fraction %)** | **Total fat (mass fraction %)** |
| --- | --- | --- |
| CSMF1 | 2.88 ± 0.01 | 2.88 ± 0.01 |
| CSMF2 | 2.88 ± 0.01 | 2.87 ± 0.01 |
| CSMF3 | 2.88 ± 0.01 | 2.93 ± 0.01 |
| CSMF4 | 2.87 ± 0.01 | 2.88 ± 0.01 |
| Semi-skimmed milk (this study) | 2.86 ± 0.01 | 2.87 ± 0.01 |
| Cocoa (10% solution) (this study) | 3.18 ± 0.01 | 9.36 ± 0.01 |
| Semi-skimmed milk (INEN 701:2009) | 2.9 | min 1.5-max 2.9 |
| Milk fermented beverages (FAO CXS-243:2003) | 2.7 | <10% |
| Cocoa powder (INEN 620:2017) | 3.2 | 8-28 (cocoa butter equivalents) |

Note: CSMF1:semi-skimmed milk + 1% cocoa + 2% glucose; CSMF2: semi-skimmed milk + 2% cocoa + 2% glucose; CSMF3: semi-skimmed milk + 3% cocoa + 2% glucose; CSMF4: semi-skimmed milk + 2% glucose;
